# 500,000 fish phenotypes: The new informatics landscape for evolutionary and developmental biology of the vertebrate skeleton

**DOI:** 10.1111/j.1439-0426.2012.01985.x

**Published:** 2012-06

**Authors:** P Mabee, J P Balhoff, W M Dahdul, H Lapp, P E Midford, T J Vision, M Westerfield

**Affiliations:** 1 Department of Biology, University of South DakotaVermillion, South Dakota, USA; 2 National Evolutionary Synthesis CenterDurham, North Carolina, USA; 3 Department of Biology, University of North Carolina at Chapel HillChapel Hill, North Carolina, USA; 4 Institute of Neuroscience, University of OregonEugene, Oregon, USA

## Abstract

The rich phenotypic diversity that characterizes the vertebrate skeleton results from evolutionary changes in regulation of genes that drive development. Although relatively little is known about the genes that underlie the skeletal variation among fish species, significant knowledge of genetics and development is available for zebrafish. Because developmental processes are highly conserved, this knowledge can be leveraged for understanding the evolution of skeletal diversity. We developed the Phenoscape Knowledgebase (KB; http://kb.phenoscape.org) to yield testable hypotheses of candidate genes involved in skeletal evolution. We developed a community anatomy ontology for fishes and ontology-based methods to represent complex free-text character descriptions of species in a computable format. With these tools, we populated the KB with comparative morphological data from the literature on over 2500 teleost fishes (mainly Ostariophysi) resulting in over 500,000 taxon phenotype annotations. The KB integrates these data with similarly structured phenotype data from zebrafish genes (http://zfin.org). Using ontology-based reasoning, candidate genes can be inferred for the phenotypes that vary across taxa, thereby uniting genetic and phenotypic data to formulate evo-devo hypotheses. The morphological data in the KB can be browsed, sorted, and aggregated in ways that provide unprecedented possibilities for data mining and discovery.

## Introduction

In response to the enormous challenge presented by the deluge of new data, biologists have embarked on a new voyage of exploration and discovery using bioinformatics. Although the emphasis has been on genomic data (Pennisi, 2011), there is growing recognition that a corresponding sea of phenomic data must also be organized and made computable in relation to genomic data. Phenotypes are the observable features of an organism such as anatomy, behavior, and the development of these traits. Mapping the genome to the phenome of an organism, and integrating such data with evolutionary changeholds a high potential for scientific discovery if the challenges of data organization and access can be overcome. Significant efforts have been mounted to integrate genetic and phenotypic information in evolutionary biology (Mabee et al., 2007a; Dahdul et al., 2010b), biodiversity (Deans et al., 2012), biomedicine (Washington et al., 2009), and agriculture (Faccioli et al., 2009).

Fish skeletal biology provides an ideal testing ground for data integration, given its rich history in comparative anatomy (Cuvier and Valenciènnes, 1846), phylogenetic systematics (Williams and Ebach, 2008), and developmental biology (Grunwald and Eisen, 2002). The purpose of this paper is to describe the informatics approach initiated by the Phenoscape project (Mabee et al., 2007a; Dahdul et al., 2010a, b) that has the potential to transform the way development and evolution of the fish skeleton are studied and understood.

## Two problems in evo-devo

Biologists working at the intersection of the fields of evolution and development face two central problems when attempting to integrate data from both fields: (i) the difficulty for an expert in the comparative anatomy and evolution of a taxon to discover candidate genes for evolutionary phenotypes, and (ii) the difficulty for an expert in the molecular development and genetics of a model organism to recover the taxonomic distribution of a particular feature across the tremendous breadth of species.

An example of first problem, the difficulty of candidate gene discovery, is illustrated by numerous publications describing the comparative skeletal anatomy of fishes. For example, Wiley and Johnson (2010) collected and reviewed morphological synapomorphies, most of them skeletal features, which are the basis for recognizing 118 major groups of teleost fishes. The first six characters described are some of the synapomorphies for teleosts and involve aspects of the quadrate, maxilla, coronoid bones, articular, neural spine of preural centrum 1, and the pectoral propterygium. Although quite laborious, a thorough literature search would yield candidate genes that have phenotypic effects on the quadrate and maxilla in zebrafish, as would a search for these bones in the ZFIN database (http://zfin.org). No genes, however, that have a phenotypic effect on the coronoid bones, articular, neural spine of preural centrum 1, or the pectoral propterygium, can be retrieved currently from these sources.

The second problem, i.e., the difficulty of recovering the distribution of a particular feature for a set of taxa, can be generally illustrated by most aspects of the fish skeleton. Of interest to a developmental geneticist might be to identify the variation across fishes of a skeletal phenotype for a particular gene, such as the *brpf1* gene that results in the loss of a basihyal cartilage when mutant in zebrafish (Laue et al., 2008). Phenotypic data on the skeletal variation of fishes, however, are recorded in an enormous bulk of free-text based literature such as books, monographs, unpublished theses, dissertations, phylogenetic studies, species descriptions, and taxonomic treatments. Although this literature is being digitized, it is still not organized or accessible for finding, aggregating, or comparing phenotypic data across studies. Even fish skeletal data that are available in databases, such as FishBase (Froese and Pauly, 2011), are not easily accessible for effective browsing, comparison, or analysis. From an Internet search of ‘basihyal lost absent fish’, one might patch together that the basihyal was lost possibly twice in batoid fishes (Miyake and McEachran, 1991), is absent in Pterygotriglini (Richards and Jones, 2002), etc. But it is not immediately obvious from the 5000+ search results that all Siluriformes (catfishes) share the derived loss of the basihyal element (Arratia and Schultze, 1990; de Pinna, 1993). Moreover, it is not possible to collect a hierarchically ordered list of fishes with associated presence or absence of basihyal element, and it is not possible to visualize the distribution of this feature on a phylogenetic tree for fishes. For even the most expert fish anatomist, recovering data on the variation in a particular feature across fishes is extremely difficult, and for students, or for researchers from other disciplines, it is completely unreachable.

## The Phenoscape project, ontologies, and phenotypes

The Phenoscape project was launched in 2007 to enable large-scale knowledge discovery in the field of evolutionary developmental biology. The aim was to solve the two fundamental problems (above) by semantically integrating data on phenotypic variation among species with the phenotypic effects of genetic variation in model organisms using shared ontologies. To date, the project has produced a proof-of-concept knowledgebase with more than 5 00 000 phenotype assertions from the ichthyological literature.

Information retrieval from free-text is difficult (Washington et al., 2009). Simple text matching will not recognize that the following four phenotypes/character states refer to the same bone: ‘lacrymal bone, flat’ (Mayden, 1989), ‘lacrimal, small, flat’ (Grande and Poyato-Ariza, 1999), ‘first infraorbital (lachrymal) shape, flattened’ (Kailola, 2004), ‘suborbital bone is very broad’ (Cuvier, 1840). However, if these text strings are annotated with the ontology identifier TAO:0000223, which corresponds to infraorbital 1 and its synonyms (lacrymal, lacrimal, first infraorbital, suborbital), and uniquely references this concept as the first or anterior-most dermal bone that is located adjacent to the orbit in fishes, these differently described phenotypes can be aggregated. Moreover, if both infraorbital 1 and infraorbital 4 are related in an ontology as types of infraorbitals, parts of the infraorbital series, and types of dermal bones, they can be returned in queries for the term ‘infraorbital’, ‘infraorbital series’, or ‘dermal bone’. Thus, free text terms that are synonymized and related in an ontology can be computationally aggregated and computed in ways that are not possible with free text alone.

An ontology can thus function to relate concepts (terms) in user-defined ways. It is a hierarchical set of well-defined terms and the logical relationships that hold between them. It represents the knowledge of a discipline, in a form that can be understood by both humans and machines. Ontologies are used for standardizing terminology within disciplines and for clarifying and improving communication across domains. Model organism communities have led successful efforts to standardize gene function descriptions (Gene Ontology, Blake and Harris, 2008) and to standardize the names of anatomical structures in model organism specific ontologies, e.g., the zebrafish anatomy ontology (Sprague et al., 2001). More recently, multispecies anatomy ontologies have been developed by the evolutionary community, including one for fishes, the Teleost Anatomy Ontology (Dahdul et al., 2010b) and one for hymenopterans (Yoder et al., 2010). As recently demonstrated by the mouse, fly, and zebrafish databases (Washington et al., 2009), ontologies support interoperability of descriptive data across databases, because, in contrast to natural language, ontologies allow computer processing of the semantic information buried in textual descriptions at a large scale.

The model organism community pioneered an ontology-based approach to represent anatomical phenotypes for the purpose of integrating mutant phenotypes across model organisms (Washington et al., 2009; Mungall et al., 2007). This Entity–Quality (EQ) syntax decomposes phenotype statements into three basic components: a phenotypic quality (Q), such as a ‘flattened’ shape; the entity that is its bearer (E), such as ‘infraorbital bone’; and the organismal entity that exhibits the phenotype, in the case of model organisms, the genotype. Phenotypes in EQ format consist of terms from ontologies for each component, and well-defined relationships (*is a*, *part of*, and *develops from*) that render them formal logic expressions (Burger et al., 2008).

The Phenoscape project adopted this ontology-based approach to represent evolutionary phenotypes described as characters and character states in the systematics literature (Mabee et al., 2007b; Balhoff et al., 2010; Dahdul et al., 2010a, b). Using anatomical and taxonomic terms from teleost-specific ontologies (Teleost Anatomy Ontology and Teleost Taxonomy Ontology) in combination with terms from a taxon-neutral quality ontology (Phenotype and Trait Ontology), we used the EQ formalism to curate all characters and states described in over 50 phylogenetic studies (published between 1981 and 2008) of teleost fishes, primarily ostariophysans but also including clupeomorphs and some euteleosts (percomorphs and salmoniforms). These studies included peer-reviewed publications, book chapters, dissertations, and a M.S. thesis (Dahdul et al., 2010a). We developed the Phenex annotation software (Balhoff et al., 2010) to support a workflow for ontology-based annotation of data from these publications. Specifically, Phenex imports character matrices, loads user-selected anatomy and quality ontologies for representation of phenotypes, and facilitates selection of taxon identifiers from a taxonomic ontology, as well as museum collection IDs for recording specimens (Balhoff et al., 2010). As in other areas, the biocuration process (Howe et al., 2008) of annotating text, here phenotype descriptions, with ontology terms is primarily manual, although efforts to partially automate the work flow are underway (e.g., Dowell et al., 2009). A total of 4 820 phenotypic characters for 2 506 fish taxa, primarily species, have been curated into EQ formalism to date, resulting in 560 485 skeletal and other anatomical phenotype annotations. In the Phenoscape KB these taxon phenotype annotations are combined with 26 934 phenotype annotations for 4 307 zebrafish genes^1^. The Phenoscape KB supports browsing, searching, and analyzing gene and phenotype annotations together. It also allows users to take advantage of the relations between ontology terms, such as subtype and parthood relations. For example, a search for ‘paired fin’ will also retrieve data that are tagged with the logical subtypes ‘pectoral fin’ or ‘pelvic fin’. Other logical relations, such as *develops from*, allow more sophisticated searching. A paired fin *develops from* a fin bud, and thus there is the potential to recover genetic and phenotypic data for fin buds from a search on paired fins. From the example above (Wiley and Johnson, 2010), candidate genes associated with the parent terms of coronoid bones, articular, neural spine of preural centrum 1, and pectoral propterygium can be proposed. The pectoral propterygium, for example, is *part of* the pectoral fin and as such is associated with all 153 genes with mutant pectoral fin (including parts) phenotypes in zebrafish.

## 5 00 000 hypotheses: candidate genes, candidate taxa

The wide variety of skeletal phenotypes among fishes are integrated across studies and with genes in the Phenoscape KB, enabling the search for candidate genes underlying evolutionary phenotypes and the query for distributions of phenotypes across taxa in gene expression and function. The shared ontologies integrate these disparate data and yield a rich set of testable hypotheses. Each of the 5 60 485 fish taxon phenotype annotations is associated with one or more genes in the KB, and thus thousands of evolutionary transitions in phenotype and gene associations may be hypothesized. The nine examples below illustrate common evolutionary changes in fish skeletal phenotypes for which candidate genes may be returned from queries (i.e., questions addressed via a software interface) to the Phenoscape KB. The numbers of phenotypes, taxa, and genes that are reported below will change as new data from the literature are curated into the ZFIN and Phenoscape KB (data below from 23 November 2011).

### Gill rakers, absent

A query to the Phenoscape KB for taxa that lack gill rakers on one or more gill arches returns 101 taxa, including Anguilliforms, and some Characiformes, Siluriformes, and Tetraodontiformes. Gill rakers are absent from all gill arches of Anguilliforms (Nelson, 2006). In zebrafish, mutationsin two genes, *eda* and *edar*, result in the absence of gill rakers (Harris et al., 2008). One could then ask, for example, ‘Are gill rakers absent in anguilliform eels because of changes in *eda, edar*, or regulation of the *eda* signaling pathway?’

### Lateral line, variation

A Phenoscape KB query for taxa that vary in some quality of their lateral line, i.e., a change in position, shape, completeness, etc., yields 815 taxa in 18 teleost orders, including, e.g., *Minytrema melanops* (the spotted sucker). Alteration in the function of seven genes in zebrafish, *erbb3b, eya1* (Whitfield et al., 1996)*, lef1* (McGraw et al., 2011)*, pcsk5a* (Chitramuthu et al., 2010)*, rog, sox10,* and *unm_m583* (Driever et al., 1996) results in abnormal lateral line phenotypes. The morpholino-based translation inhibition in *pcsk5a*, for example, disrupts formation of the lateral line, resulting in reduced or complete absence of posterior lateral line neuromasts (Chitramuthu et al., 2010). This query motivates the hypothesis that reduced length of the posterior lateral line in *Minytrema* (Smith, 1992) is due to an alteration in function of *pcsk5a* (or any of the other genes above).

### Caudal fin, absent

A caudal fin is primitively present in teleost fishes; lost only a few times during evolution. A query in Phenoscape KB for absence of a caudal fin returns 28 taxa, including 25 species of gymnotiform knifefishes (Albert, 2001) and three tetraodontiform species (Santini and Tyler, 2002). The loss of the caudal fin in the familiar *Mola mola* has been confirmed through a detailed developmental morphological study (Johnson and Britz, 2005). Five genes, *edar* (van Eeden et al., 1996)*, lef1* (McGraw et al., 2011) *smc3*, *tll 1* (Lele et al., 2001) and *yap1* (Jiang et al., 2009), are associated with caudal fin loss in zebrafish. One might investigate, then, whether the loss of the caudal fin in *Mola mola* is related to changes in regulation of *yap1* (or any of the other genes above).

### Ceratobranchial five teeth, absent

A query for taxa that vary in the presence of teeth on their fifth ceratobranchial element results in 43 taxa, including some Characiformes, Gymnotiformes, Tetraodontiformes, and *Gyrinocheilus*. Three genes, *acvr2a* (Albertson et al., 2005), *eda*, and *edar* (Harris et al., 2008), when disrupted, result in phenotypes that include absence of teeth on the ceratobranchial five element in zebrafish. One might ask, for example, whether the loss of ceratobranchial five teeth in the common aquarium fish, the algae-eater *Gyrinocheilus,* is related to changes in regulation of one of these genes.

### Dorsal fin, absent

A query for taxa that lack a dorsal fin yields 58 taxa, including all Gymnotiformes (knifefishes) and some Siluridae (catfishes). Two genes, *hoxa13a* (Crow et al., 2009) and *tfap2a* (Li and Cornell, 2007), are linked to zebrafish phenotypes of median fin fold absence. Because the formal *develops from* relation relates dorsal fin to median fin fold in the Teleost Anatomy Ontology, a search for genes associated with dorsal fin phenotypes can return genes associated with the median fin fold phenotypes as well. Thus genes influencing the development of the precursors of structures may be considered as candidates in a search for the basis of the evolutionary novelty of dorsal fin loss in, for example, a gymnotiform knifefish.

### Preopercle, shape

The preopercle varies in shape in 546 taxa, including Amiiformes, Aspidorhynchiformes, Characiformes, Clupeiformes, Cypriniformes, Ellimmichthyiformes, Elopiformes, Esociformes, Gonorynchiformes, Gymnotiformes, Hiodontiformes, Lepisosteiformes, Osteoglossiformes, Salmoniformes, Semionotiformes, and Siluriformes. No phenotypes are recorded for preopercle shape in zebrafish. However, if the search is broadened to include shape phenotypes from other parts of the opercular series (branchiostegal rays, interopercle, opercle, preopercle, and subopercle) then nine genes with mutant phenotypes are revealed: *acvr2a, acvr2b* (Albertson et al., 2005)*, edn1* (Walker et al., 2007), *furina* (Walker et al., 2006), *jag1b, notch2* (Zuniga et al., 2010), *mef2ca* (Kimmel et al., 2003), *plcb3* (Walker et al., 2007), and *unm_t3153*. On the other hand, if the search is broadened from preopercle to include shape changes in other components of the dorsal hyoid arch, ten genes, some of them different from above, are found. Any of these genes provide a starting point for investigating the possible bases for shape changes in this dermal bone.

In fact, much of the skeletal variation that exists among fish species involves changes in shape, and approximately a fifth of the species phenotypes in the KB reflect this. Mutations in 920 of the 4307 zebrafish genes in the KB produce a change in shape of some aspect of anatomy in 1435 (of 26 934 total) gene phenotypes. Changes in shape, especially in integrated skeletal structures may be very indirect, reflected in the increase in number of potential links. This lack of direct causality makes hypothesis testing difficult for complex structures.

### Eye, decreased in size

A query of the Phenoscape KB informs the user that in all Siluriformes and Gymnotiformes, generalized from 57 studied species, the eye is reduced in size relative to the surrounding infraorbital bones (Fink and Fink, 1981). Mutations in 574 zebrafish genes produce reduced eye size, including, for example, *pbx4* (French et al., 2007).

### Scales, absent

Scales are frequently lost on the head and body of fishes, and Phenoscape KB lists 336 taxa from 27 teleostean orders, including e.g., Siluriformes (catfishes), in which all scales are absent except for bony tubes of the lateral line (Fink and Fink, 1981). Three zebrafish genes, *eda*, *edar* (Harris et al., 2008), and *unm_t31273*, are candidates for this phenotype.

### Basihyal, absent

All Siluriformes (catfishes) and four other teleostean species with data in the Phenoscape KB have lost the basihyal element, i.e., the anterior-most median element of the gill arches, or ‘tongue’ of the fish. The disruption of eleven zebrafish genes, including *brpf1* (Laue et al., 2008), *disc1* (Wood et al., 2009), *disp1* (Schwend and Ahlgren, 2009), *fac* (Schilling et al., 1996), *foxd3* (Neuhauss et al., 1996), *hand2* (Miller et al., 2003), *kat6a* (Laue et al., 2008), *sox9a* (Yan et al., 2002), *unm_th9*, *unm_tn20c*, and *unm_ty5* result in an aplastic or absent basihyal phenotype.

## Limitations and biases in candidate genes –taxon approach

These examples demonstrate the ease with which genetic phenotypes may be aligned with diverse taxon phenotypes using ontologies to yield testable hypotheses. They also expose possible limitations, such as a bias toward well-studied pleiotropic genes such as *eda* or *edar*, which are proposed as candidates in several of the above cases. Missing phenotype data for taxa, whether because the taxa have never been surveyed for particular features or because these data have not been entered into the Phenoscape KB, also limit this approach, as does the coarseness of phenotype annotations for both genes and taxa. Other sources of bias include the different focus of phenotypic study in zebrafish developmental biology (neural system) vs. fish comparative morphology (skeletal system) and the difference in developmental stage under study, i.e., embryos and larvae in zebrafish vs. adults in comparative studies.

## Integration of taxon phenotypes across studies

**Fig. 1:**
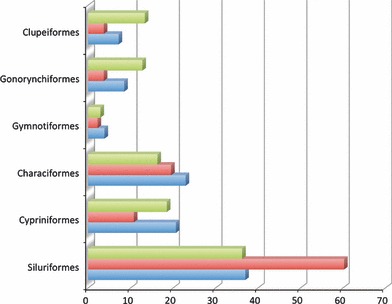
Distribution of skeletal phenotypes in the Phenoscape Knowledgebase across the cranium (blue), paired fins (red), and postcranial axial skeleton (green) for Clupeiformes and the five ostariophysan orders

Ontology-annotations of phenotypic data surmount the difficulty of recovering the distribution of any skeletal feature across a set of taxa. The data can easily be viewed, summarized, and synthesized across studies at a scale not previously possible. As an example, the distribution of skeletal data, stratified by skeletal region across the five orders of ostariophyan fishes and their sister taxon order, the Clupeiformes (Fig. 1) reveals the disproportionate level of data on paired fins in the Siluriformes, reflecting the rich variation in pectoral fin spine ornamentation in this clade. The distribution of species phenotypes across particular skeletal elements, body regions, etc. can be ascertained easily for any sample of publications in the KB.

## Translational biodiversity

One of the most significant discoveries from the past decade is that genes, intron–exon structure, synteny, gene expression patterns, networks, and developmental processes, are highly conserved, well beyond expectation, across very distantly related organisms (Degnan, 2010). Examples of deep conservation or ‘deep homology’ (Shubin et al., 2009) have become common and familiar across greatly divergent species, e.g., the function of *Irx* and *hnf1* genes in lampreys are conserved across vertebrates for positioning the r4/r5 boundary (Jimenez-Guri and Pujades, 2011); the pigment gene *slc24a5*is functionally conserved between zebrafish and humans (Lamason et al., 2005); common genes control eye development (Gehring, 2004) and appendage development (Pueyo and Couso, 2005) from insects to humans (Carroll et al., 2005).

Biomedical researchers have leveraged this conservation to translate studies from model organisms to human medicine, so-called ‘translational medicine’ (Washington et al., 2009). For example, because important pathways such as insulin signaling have remained relatively unchanged during evolution, many human diseases, including cancer, are studied effectively in the nematode *C. elegans* (Markaki and Tavernarakis, 2010). Human craniofacial defects have been elucidated significantly by studies of zebrafish (Ghassibe-Sabbagh et al., 2011; Laue et al., 2011; Petrey et al., 2011), mouse (Tuveson and Hanahan, 2011), and *Xenopus* (Warkman and Krieg, 2007).

Further leveraging the conservation of developmental genes and networks to translate from model organisms to the breadth of species beyond humans, an approach that could be termed ‘translational biodiversity,’ is supported by the Phenoscape KB. Here, and demonstrated by the examples above, genetic and developmental data from zebrafish are leveraged to propose candidate genes for evolutionary changes in skeletal phenotypes across fish species, and the descriptive data from the field of comparative and evolutionary morphology are made accessible for searching and aggregating data across the breadth of species. These two disparate data-stores, one from molecular genetics and development and the other from comparative morphology and evolution, can thus be rendered explorable and usable to researchers in other domains.

Although some differences in developmental mechanisms among similar phenotypes will certainly have arisen in the course of evolution (e.g., Tanaka et al., 2002), this computational approach to generating candidate genes is attractive, because studying the genetic and developmental bases of evolutionary phenotypes using the laboratory approaches from model organisms is simply impractical for the millions of extant species on earth. A fully developed ‘translational biodiversity’ will require databases of computable phenotypes for both model organisms and taxonomic groups that computers can understand and reason across. Such an ontology-based approach promises powerful data synthesis and discovery at a scale not otherwise possible. It also makes data accessible for broad groups of researchers and creates opportunities for new and synthetic research.

## Footnotes

1 Numbers of phenotypes, taxa, and genes annotated change as new data are curated into the ZFIN and Phenoscape KB.
